# An analysis of the regional heterogeneity in tissue elasticity in lung cancer patients with COPD

**DOI:** 10.3389/fmed.2023.1151867

**Published:** 2023-09-28

**Authors:** Michael Lauria, Bradley Stiehl, Anand Santhanam, Dylan O’Connell, Louise Naumann, Michael McNitt-Gray, Ann Raldow, Jonathan Goldin, Igor Barjaktarevic, Daniel A. Low

**Affiliations:** ^1^Department of Radiation Oncology, University of California, Los Angeles, Los Angeles, CA, United States; ^2^Department of Radiological Sciences, University of California, Los Angeles, Los Angeles, CA, United States; ^3^Division of Pulmonary and Critical Care Medicine, University of California, Los Angeles, Los Angeles, CA, United States

**Keywords:** COPD, elasticity, lung heterogeneity, biomechanical properties, function sparing treatment planning

## Abstract

**Purpose:**

Recent advancements in obtaining image-based biomarkers from CT images have enabled lung function characterization, which could aid in lung interventional planning. However, the regional heterogeneity in these biomarkers has not been well documented, yet it is critical to several procedures for lung cancer and COPD. The purpose of this paper is to analyze the interlobar and intralobar heterogeneity of tissue elasticity and study their relationship with COPD severity.

**Methods:**

We retrospectively analyzed a set of 23 lung cancer patients for this study, 14 of whom had COPD. For each patient, we employed a 5DCT scanning protocol to obtain end-exhalation and end-inhalation images and semi-automatically segmented the lobes. We calculated tissue elasticity using a biomechanical property estimation model. To obtain a measure of lobar elasticity, we calculated the mean of the voxel-wise elasticity values within each lobe. To analyze interlobar heterogeneity, we defined an index that represented the properties of the least elastic lobe as compared to the rest of the lobes, termed the Elasticity Heterogeneity Index (EHI). An index of 0 indicated total homogeneity, and higher indices indicated higher heterogeneity. Additionally, we measured intralobar heterogeneity by calculating the coefficient of variation of elasticity within each lobe.

**Results:**

The mean EHI was 0.223 ± 0.183. The mean coefficient of variation of the elasticity distributions was 51.1% ± 16.6%. For mild COPD patients, the interlobar heterogeneity was low compared to the other categories. For moderate-to-severe COPD patients, the interlobar and intralobar heterogeneities were highest, showing significant differences from the other groups.

**Conclusion:**

We observed a high level of lung tissue heterogeneity to occur between and within the lobes in all COPD severity cases, especially in moderate-to-severe cases. Heterogeneity results demonstrate the value of a regional, function-guided approach like elasticity for procedures such as surgical decision making and treatment planning.

## 1. Introduction

Respiratory diseases are a major cause of death, including Chronic Obstructive Pulmonary Disease (COPD), which leads to over 3 million deaths annually (6% worldwide), making it the third leading cause. More than 90% of these deaths occur in low-income or middle-income countries (1). Based on a meta-analysis conducted over studies from 1960 to 2010, COPD is associated with an increased risk of lung cancer with a hazard ratio of 2.22 (2). In addition to the heightened risk of cancer with COPD, one study showed that the cancer mortality rate is also greatly increased with increasing COPD severity, with the worst hazard ratio being 3.36 for GOLD stage 4 (3).

Since COPD is a notoriously heterogeneous disease (4–8), several pulmonary procedures for patients with COPD exploit regional lung function differences. One surgical treatment that has been developed for patients with severe COPD is lung volume reduction surgery (LVRS) (9). When a lobe experiences reduced ventilation due to air trapping, LVRS aims to collapse the lobe and release the trapped air, thus allowing the other more functional lobes to compensate (10, 11). An additional surgical technique building off LVRS is bronchoscopic lung volume reduction (BLVR), in which a one-way collapsible coil is placed in the airway (12, 13). LVRS, BLVR, and other lung interventions depend on disease heterogeneity to ensure that lung function can be compensated post-intervention (14, 15). Not only has heterogeneity been specifically identified as a factor in patient selection for these surgeries, but further research into patient selection, physiological testing, and disease characteristics have been called for in the literature (11, 13, 16).

In addition to surgical interventions, radiation therapy is another example of a treatment that could benefit from an understanding of the regional functionality of lung tissue, specifically when sparing organs at risk (OARs). A recent study showed that patients with lung cancer and COPD receive less curative treatments and experience higher mortality rates with a hazard ratio of 1.2 (17). In addition to poorer survival rates, normal tissue complications have also been observed in lung cancer patients with COPD comorbidities (18, 19). These increased risks of mortality and side effects call for a heightened attention to the regional effects of COPD in treatment planning. This information could aid functionally guided organs at risk (OARs) as opposed to the current lung-based contours (20–22). Studies using CT-based or PET-CT-based ventilation for functional tissue sparing have already shown reduction in doses to functional tissue (23–25) as well as reduction in grades 2+ and 3+ pneumonitis (24, 26).

To diagnose regional functionality, ventilation has been previously employed as a key biomarker. Evidence has been shown that ventilation mapping could aid in treatment planning by highlighting areas of high and low ventilation (27). While these studies of functional tissue mapping using ventilation are promising, accurately calculating ventilation from CT is still an ongoing effort (28–31). Many of the developed approaches incorporate transformation-based calculations, or calculations based on a mapping of CT images and comparison of densities, both of which rely on the accuracy of deformable image registration. It has been shown that the even small errors in the image registration can cause much larger errors in ventilation calculations (32, 33). For this reason, it may be beneficial to explore other functional properties to guide surgeries or radiotherapy until image registration can be performed with sufficient accuracy.

We propose elasticity as an additional functional property that can be measured from CT. Tissue elasticity is a biomechanical property that describes tissue stiffness (34). Elasticity can be calculated from medical images in several ways. In our work, it is done so with the anatomy from an end-exhalation image and the deformation vector field (DVF) mapping it to end-inhalation. These inputs are provided to an iterative model that estimates the Young’s modulus (YM) of each voxel to represent the elasticity, as has been validated in previous work (34, 35). Though also based on image registration, elasticity can be reliably calculated on a voxel-by-voxel basis since it is regularized by a physics-based model. Reduced elasticity is indicative of lung disease, so it can be another regional marker for COPD. Previous work showed that elasticity of voxels in the 1–3 kPa range was a better biomarker for COPD than the traditional RA950 (36). CT-based elasticity measurements thus offer an additional way to characterize regional lung function based on CT and a physics-based model.

In this study, we statistically characterized the regional lung function heterogeneity in patients with lung cancer and varying COPD comorbidities using tissue elasticity. Lobar elasticity distributions were calculated using our inverse biomechanical model. Interlobar heterogeneity was analyzed to provide information specifically relevant to surgical interventions. Additionally, intralobar heterogeneity was analyzed to provide insight to support regionally defined treatments such as radiotherapy. We related each heterogeneity measure to COPD severity. By providing evidence of tissue elasticity heterogeneity, we have shown evidence of how it could benefit surgical or radiotherapy planning for patients with all levels of COPD.

## 2. Materials and methods

**Figure 1:**
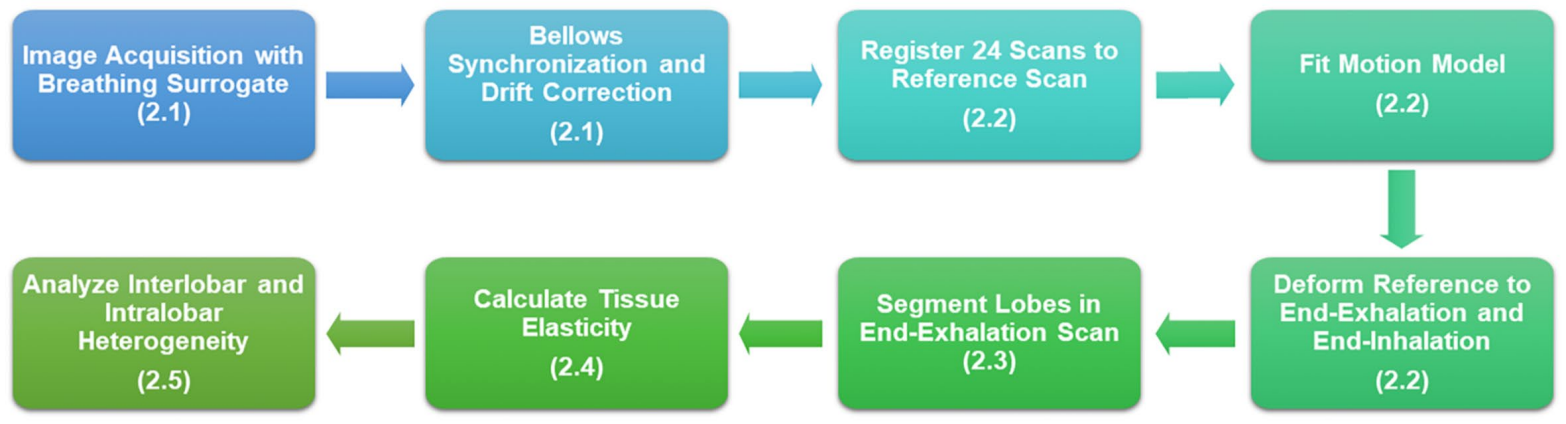
Flowchart of the study design beginning with image acquisition and resulting in heterogeneity analysis of tissue elasticity.

We used a dynamic imaging protocol to obtain all patient data in this study. We acquired fast-helical free-breathing CTs (FHFBCT), constructed motion models using the 5DCT approach, and generated end-exhalation and end-inhalation images to serve as input for our tissue elasticity estimation model. Each step is outlined in Figure 1 with the relevant Materials and Methods sections labeled.

### 2.1. Patient data acquisition

We retrospectively employed a set of 23 lung cancer patients for this study. Each patient was identified as having no COPD, mild COPD, or moderate-to-severe COPD as defined by their physicians and based on spirometry. These severities were noted based on a retrospective review of their charts. Five patients had mild COPD and six had either moderate or severe COPD. Three patients were noted to have COPD, though the severity was unknown from their charts. These patients were only included in the analysis when examining all COPD patients.

For each patient, we collected a set of 25 FHFBCTs through an IRB approved study, as in previous investigations (37, 38). The scans were acquired in alternating directions with 120 kVp and 40 mAs. Three similar multi detector row CT scanners were used (Definition Flash, Biograph 64, Definition AS 64; Siemens Healthcare, Erlangen, Germany). Table 1 summarizes the scanning parameters. For all images, the field of view was set to 500 mm. The in-plane pixel resolution was 0.976 × 0.976 mm, and the slice thickness was 1.0 mm. After reconstruction, we resampled all images to 1 mm isotropic voxels.

**Table 1:** Summary of scanners used to acquire patient data.

Scanner	Rotation period (s)	Pitch	Irradiation time (s)	Table speed (mm/s)	Scan time (s)	Delay between scans (s)	Total acquisition time (s)
Definition flash	0.285	1.2	0.238	161.4	2.5	2	140
Biograph 64	0.330	1.5	0.220	87.02	4.5	6	275
Definition AS 64	0.330	1.5	0.220	87.02	4.5	3	200

The FHFBCT acquisition process also included a simultaneous acquisition of breathing amplitude and flow rate (time-derivative of the amplitude) signals, which were needed for the 5DCT model construction to generate the end-exhalation and end-inhalation images. The breathing amplitude signal was obtained by using a pneumatic bellows (Lafayette Instrument Company, Lafayette, IN). The bellows was placed around the abdomen since its expansion was observed to provide the best correlation to the diaphragm motion (37). The bellows converted the change in pressure resulting from expansion into a voltage signal. The signal was sampled at 100 Hz, and amplitudes were assigned to each transverse slice. The bellows signals were finally synchronized and drift-corrected to account for measurement-related errors as previously demonstrated in O’Connell et al. (39).

### 2.2. 5DCT modeling

5DCT is a model-based CT approach that has been well-validated in previous works (38, 40–42). The 5DCT modeling process used in this study is briefly explained as follows.

The model generation process takes as input the 25 FHFBCT scans and the breathing signal amplitudes and flow rates. From the 25 FHFBCT images, we arbitrarily chose the first scan as the reference for the 5DCT model construction. Using an open-source deformable image registration software, deeds (43–45), the other 24 images were deformably registered to the reference scan as previously demonstrated (46, 47). We used the 24 DVFs with the breathing amplitude, v , and rate, f , to determine tissue-specific motion parameters, $\vec{\alpha }$ and $\vec{\beta }$ , by solving the relation shown below.


(1) $$\vec{X}=\vec{X_{0}}+\vec{\alpha }v+\vec{\beta }f$$

In Equation 1, $\vec{X_{0}}$ describes the tissue position at zero amplitude and flow, and $\vec{X}$ describes the tissue position at v and f . The inhalation motion is represented by the product of $\vec{\alpha }$ and the amplitude. Similarly, the hysteresis motion is represented by the product of $\vec{\beta }$ and the breathing rate.

To perform lobar HU-based and biomechanical property measurements, end-exhalation and end-inhalation images along with their corresponding DVFs needed to be generated. We selected the 5^th^ and 85^th^ percentile amplitudes with zero flow to represent end-exhalation and end-inhalation respiratory phases as shown in previous studies (42). Using Equation 1, the tissue-specific motion parameters were used to deform the reference image to its position in the end-exhalation and end-inhalation breathing phases.

### 2.3. Lobe segmentation

To obtain lobar elasticity distributions, we generated lobe masks that grouped lung voxels into one of the five lung lobes. We performed lobe segmentations semi-automatically on the FHFBCT reference scans using the open-source software Pulmonary Toolkit. The software first built a lobar approximation, then applied a “fissureness” filter, and finally fit a smooth multi-level B-spline curve through the fissureness and extrapolated to the lung boundaries to create the lobe segmentation (48). In some cases, the automated segmentation results experienced minor errors, so manual corrections were made using the graphical user interface in the Pulmonary Toolkit and verified by medical experts.

To only include lung parenchyma in the analysis, we removed blood vessels and tumors from the lobe masks by excluding voxels with greater than −700 HU. This threshold was chosen based on the HU distribution found in the lungs of clinical CT scans in a published study (49). Though this reference used inspiration CT rather than free-breathing CT, we experimented with higher thresholds and −700 HU was optimal for removing all vessels from the images. This is important because blood vessels do not expand or ventilate during respiration and have very high elasticity, so their values would misrepresent the lung parenchyma distributions. Therefore, our analysis only pertained to the parenchymal tissue with HU less than −700 in the 5th percentile images.

### 2.4. Tissue elasticity estimation

We used a previously developed and validated biomechanical model to estimate elasticity of the lung parenchyma, evaluated as the YM. The biomechanical model is based on changes in boundary constraints leading to corrective forces on a distribution of finite elements. These corrective forces are a summation of elastic, shear, and dashpot damping forces, which are included in Equations 2–4, respectively. In these equations, YM is Young’s Modulus, $\Delta L_{ab}$ is the change in length between elements a and b , $L_{ab}$ is the resting length between a and b , $S_{ab}$ is the shear moduli (4 kPa), $\mu_{ab}$ is the local damping factor, and $\vec{v}$ is relative velocity.


(2) $$\vec{f_{E,ab}}=\underset{b}{\sum }\left(YM*\frac{\Delta L_{ab}}{L_{ab}}\right)\;$$

(3) $$\vec{f_{2S,ab}}=\underset{b}{\sum }\left(S_{ab}*\frac{L_{ab}-\Delta L_{ab}}{L_{ab}}\right)\;$$

(4) $$\vec{f_{v,ab}}=\underset{b}{\sum }\left(\mu_{ab}{}^{*}\left(\vec{v_{b}}-\vec{v_{a}}\right)\right)\;$$

**Figure 2:**
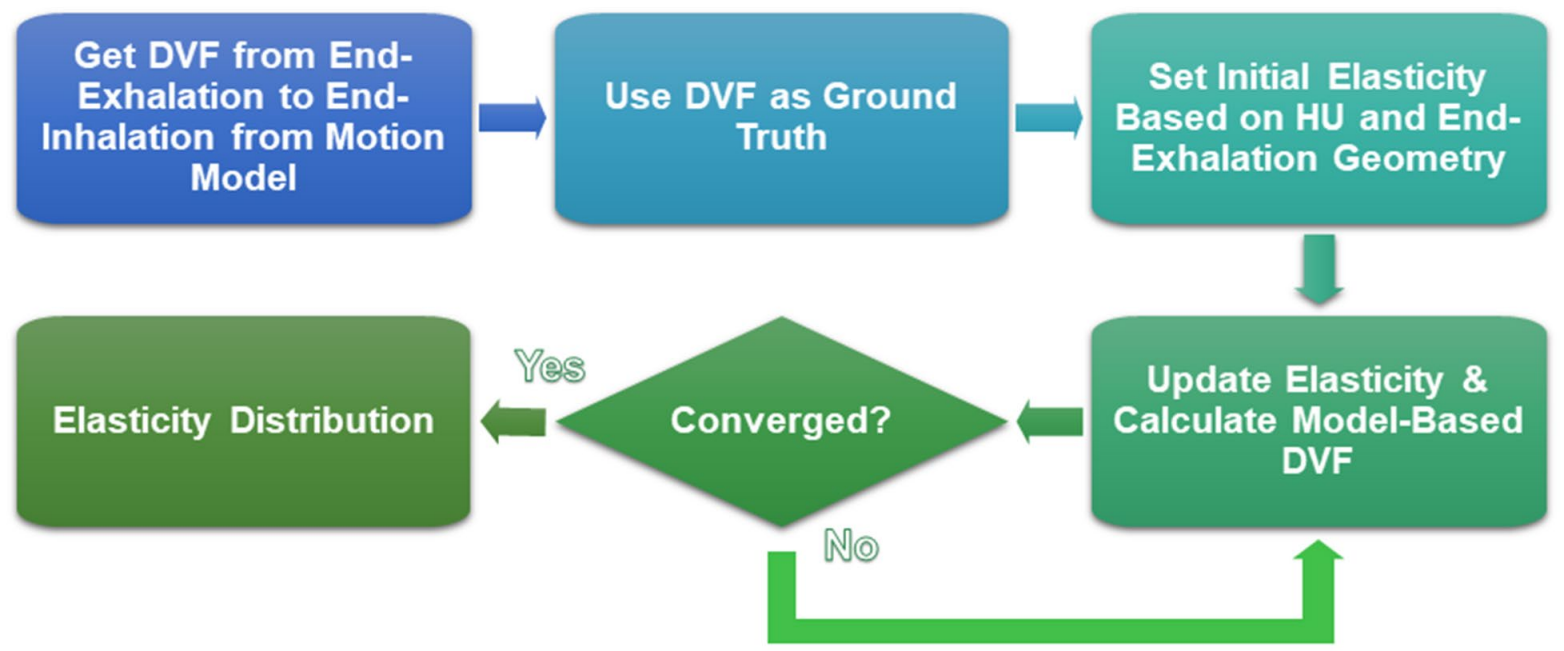
Flowchart of the iterative parameter optimization problem to estimate elasticity.

In this study, the model used the DVF pointing from the 5DCT-based end-exhalation image to the end-inhalation image as the ground-truth DVF. Then, an initial elasticity distribution was set based on the HU of the end-exhalation scan. The elasticity distribution was then optimized to minimize the difference between the model-calculated DVF, based on boundary conditions and the calculated deformations from elastic forces, and the ground-truth DVF. In each iteration, the elasticity was updated, the new DVF was calculated from the updated elasticity values, and the DVF was compared to the ground truth. This process is shown in the flowchart in Figure 2. Further details of the finite element approach, governing equations, boundary conditions, and description of the inverse approach to optimize the elasticity can be found in several publications describing the model (34, 35, 50). The lobar elasticity was obtained by calculating the mean elasticity across each lobe distribution.

### 2.5. Heterogeneity analysis

First, we analyzed the mean elasticity of each lobe for patients in each COPD severity group. This was to investigate how well elasticity represented COPD severity in our cohort, as well as to observe the lobar trends in elasticity.

To measure interlobar heterogeneity, we defined the Elasticity Heterogeneity Index (EHI). According to a previous study, elasticities in the range of 1–3 kPa indicated diseased lung because COPD causes the lungs to poorly respond to expansion or contraction (36). Therefore, we based the EHI on the maximum percent of voxels in the COPD biomarker range among the five lobes, $E_{\max}$ , and the mean of the other four lobe percentages, $\bar{E_{\sim \max}}$ . This index indicated the least elastic lobe compared to the other four while emphasizing diseased tissue. We compared the mean EHI across COPD severity groups to study the interlobar heterogeneity in elasticity for these patients.


(5) $$EHI=\left|1-\frac{100-E_{\max}}{100-\bar{E_{\sim \max}}}\right|$$

To measure intralobar heterogeneity in elasticity, we calculated the coefficient of variation, or the standard deviation as a percent of the mean, of the elasticity distributions within each lobe. This metric summarized the spread of data within each lobe distribution. We examined histograms of the coefficient of variation across all lobes of patients in each COPD severity group to study differences in interlobar elasticity heterogeneity.

The coefficient of variation offers information about the dispersion of the data. To investigate the range of elasticity in each scenario, we also calculated an index of non-uniformity. This value has been defined slightly differently across the literature, but we have modified a definition from Jadhav, et al. because it offers a good sense of the range of any given metric (51). Our non-uniformity index is defined in Equation 6, where NU is the non-uniformity index, $E_{95^{th}}$ is the 95th percentile elasticity, and $\bar{E}$ is the mean elasticity.


(6) $$NU=\frac{E_{95^{th}}-\bar{E}}{\bar{E}}$$

We performed two sample *t*-tests to test the differences between different COPD severity groups for each previously mentioned parameter. We used an F-test for variance equality to determine if the *t*-tests should be tested with or without equal variances. We tested the differences between these groups in elasticity, EHI, and intralobar coefficient of variation at the 5% significance level.

### 2.6. Tumor presence

Since all of these patients had lung cancer, there was always at least one lobe that contained a tumor. To explore the effect of the tumor on our analysis, we first separated the lobe(s) with the tumor (five of the patients had two lobes containing tumors) from the other non-cancerous lobes for each patient and calculated the mean elasticity of each group of lobes. We calculated the mean of these differences across patients as well as the mean difference relative to the mean elasticity of all five lobes. We also performed a two sample *t*-test for each patient to see if the lobe with the tumor had a significantly different elasticity than the other lobes.

However, lobe-dependent trends in elasticity may mask this effect and render interpretation of these results difficult. Therefore, we also calculated how often the lobe with the highest elasticity was also the lobe containing the tumor. We also tested across patients while keeping the lobe constant to see if the group with the tumors had a significantly different elasticity than the group without the tumors. For example, testing if the right upper lobes with tumors were significantly different than the right upper lobes without tumors.

## 3. Results

### 3.1. Elasticity

**Figure 3:**
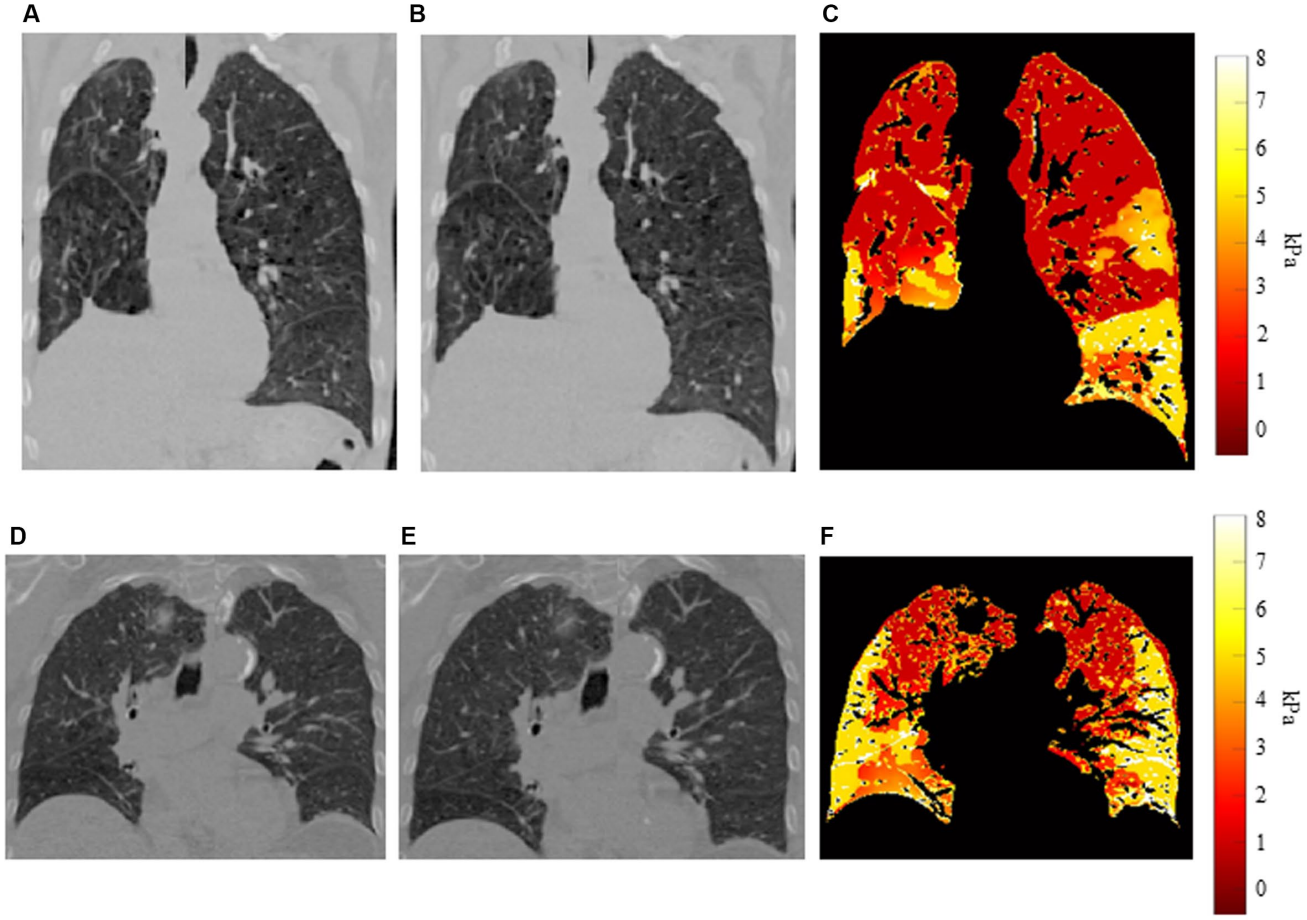
Example distributions for two patients **(A)** End-exhalation scan, **(B)** end-inhalation scan, and **(C)** elasticity distribution for patient with high interlobar heterogeneity. **(D)** End-exhalation scan, **(E)** end-inhalation scan, and **(F)** elasticity distribution for patient with low interlobar heterogeneity.

Figure 3 shows examples of our results for two patients. Figures 3A,B show coronal slices of the end-exhalation and end-inhalation scans, respectively, for the patient with high interlobar heterogeneity (EHI = 0.560). The pronounced elasticity difference between the left upper and left lower lobes is apparent in the elasticity distribution shown in Figure 3C. Figures 3D,E show the end-exhalation and end-inhalation scans for a patient with lower interlobar heterogeneity (EHI = 0.248). Regions of high and low elasticity are more dispersed throughout the lobes in this case.

**Figure 4:**
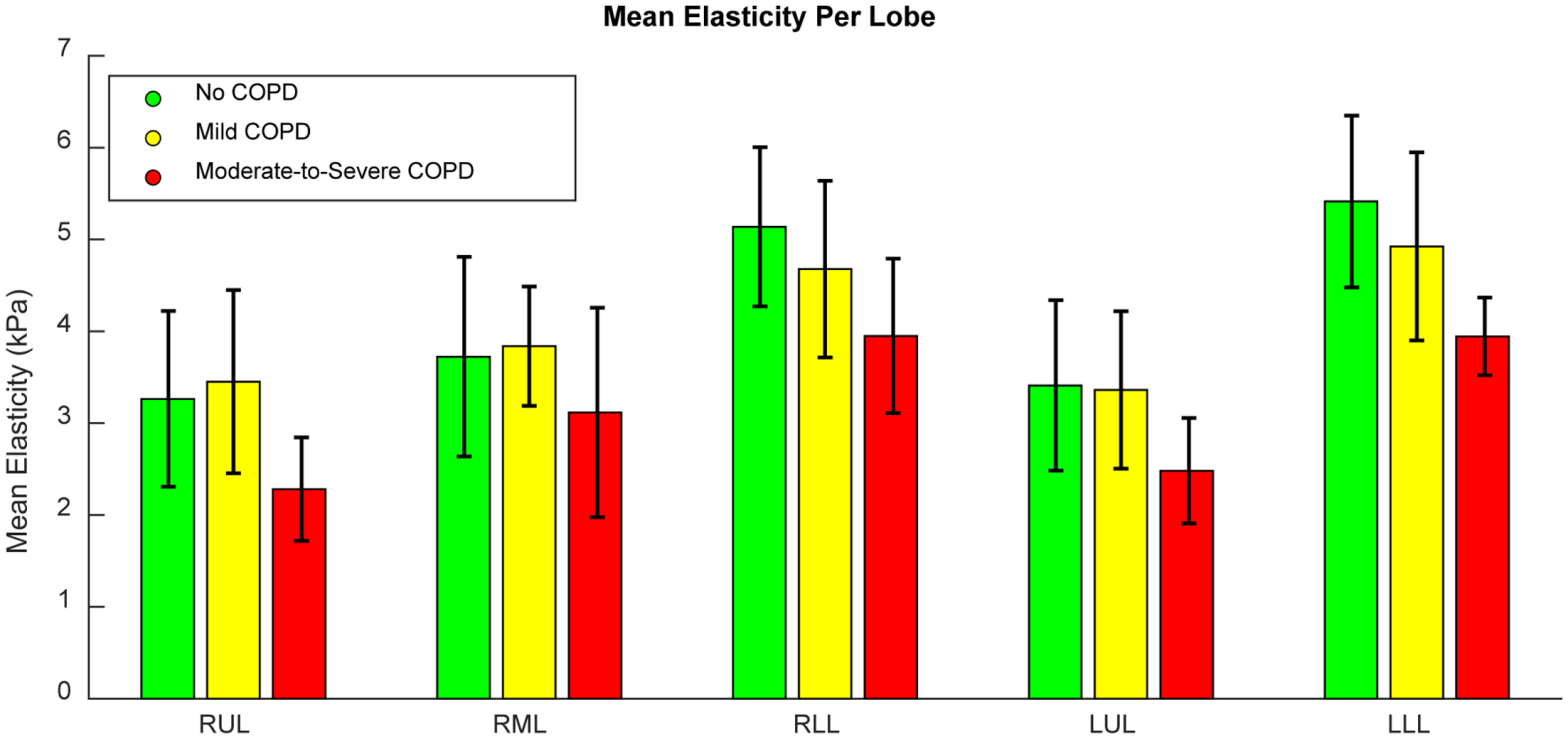
Bar graph of the mean lobar elasticity of patients in each COPD severity group. Error bars indicate standard deviations.

Figure 4 shows a bar graph of the mean elasticity across patients in each COPD severity group separated into lobes, with error bars denoting the standard deviations. It is notable that elasticity was greatest in the lower lobes, which is reflective of their larger deformations during inhalation. This figure not only shows the typical elasticity differences between lobes, but also shows that elasticity decreased for patients with moderate-to-severe COPD, though the error bars still overlapped. When comparing the mean elasticity of the entire lungs across severity groups, significant differences were seen between patients with moderate-to-severe COPD and patients without COPD (*p* < 0.01), patients with mild COPD (*p* < 0.01), and all patients (*p* < 0.01). Significant differences were seen between patients with COPD and without COPD as well (*p* = 0.02), which was most likely heavily weighted by the patients with moderate-to-severe COPD. No statistically significant differences were seen between patients with mild COPD and patients without COPD. All comparisons were found to have equal variances as a result of F-tests. Overall, we found that the effect of COPD on tissue elasticity was most prominent in moderate-to-severe cases.

### 3.2. Heterogeneity

The mean EHI was 0.388 ± 0.162. Therefore, on average, there were moderate levels of interlobar heterogeneity with a relatively high level of variation across the patient cohort. For patients with no COPD, mild COPD, and moderate-to-severe COPD, the mean EHIs were 0.385 ± 0.183, 0.258 ± 0.133, and 0.473 ± 0.128, respectively. The greatest degree of interlobar heterogeneity was seen in patients with moderate-to-severe COPD. All of these patients had EHIs of at least 0.2. Additionally, among patients with mild COPD, EHIs varied greatly. The mean EHI in patients with mild COPD was lower than those without COPD, though the difference was not statistically significant (*p* = 0.20). However, the difference in EHI between patients with mild COPD and patients with moderate-to-severe COPD was statistically significant (*p* = 0.02). All comparisons were found to have equal variances as a result of F-tests.

**Figure 5:**
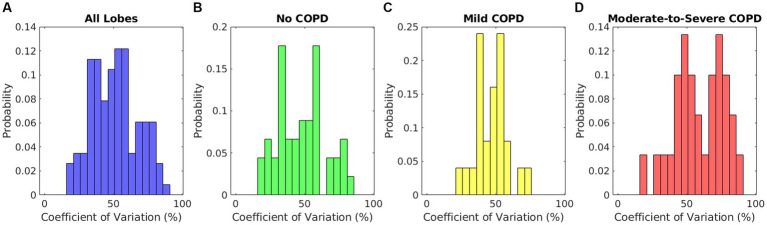
Relative histograms of the coefficients of variation of elasticity distributions for **(A)** lobes of all patients, **(B)** lobes of patients without COPD, **(C)** lobes of patients with mild COPD, and **(D)** lobes of patients with moderate-to-severe COPD.

The coefficients of variation of each lobar elasticity distribution are summarized by the histograms in Figure 5. The histogram in Figure 5A includes all lobes to offer a general sense of intralobar heterogeneity regardless of disease state. The mean of this distribution was 51.1% ± 16.6%, showing that there was a high level of intralobar heterogeneity in general. Figure 5B shows the histogram of the coefficient of variation for the lobes of patients without COPD (mean 47.9% ± 17.5%), and Figure 5C shows the histogram for lobes of patients with mild COPD (mean 47.3% ± 11.2%). These two groups exhibited a similar distribution to each other and to the distribution of all patient lobes. On the other hand, the histogram of the moderate-to-severe COPD group coefficients shown in Figure 5D exhibited a higher distribution (mean 58.9% ± 17.7%). The only significant differences were between the coefficient of variation of lobes with moderate-to-severe COPD and each of the other groups (*p* = 0.01 when comparing to lobes without COPD, *p* < 0.01 when comparing to lobes with mild COPD, *p* = 0.03 when comparing to all lobes). All comparisons to patients with mild COPD were tested with unequal variances as determined by F-test results, indicating that patients with mild COPD may have much more variation in their intralobar heterogeneity. All other comparisons were tested with equal variances. This indicates that patients with more severe COPD may experience higher levels of intralobar heterogeneity, which may be potentially useful as a metric for function-preserving interventions.

**Figure 6:**
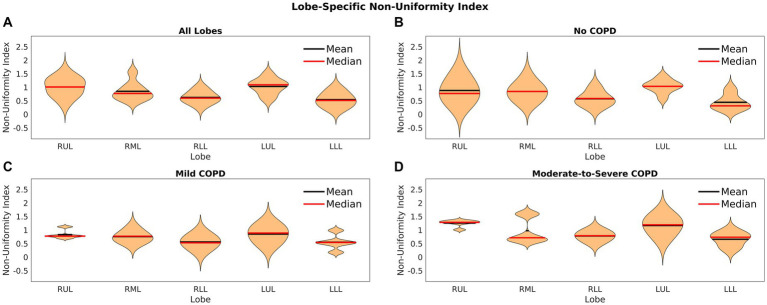
Violin plots of the index of non-uniformity for **(A)** lobes of all patients, **(B)** lobes of patients without COPD, **(C)** lobes of patients with mild COPD, and **(D)** lobes of patients with moderate-to-severe COPD.

Figure 6 shows violin plots of the non-uniformity index across the five lobes for all patients as well as each COPD severity group. Figure 6A offers a sense of the non-uniformity across the entire patient cohort. The mean value was about 1 for each group and lobe, which shows that the highest elasticity was most often twice the mean value. Figure 6 also shows that in all COPD severity groups, the index of non-uniformity was slightly less in the lower lobes. In some cases, the non-uniformity index was as high as 2.5, showing that the elasticity could be 3.5 times the mean in some regions of the lungs. These results show a high degree of heterogeneity expressed as the range of elasticity among all patients.

### 3.3. Tumor presence

Comparing the elasticity of the lobe(s) with the tumor to the mean of the elasticity of the non-cancerous lobes, we found that the mean of the absolute differences between these values across all patients was 1.011 ± 0.470 kPa. The mean of the elasticity differences relative to the mean elasticity of all five lobes was 27.5% ± 15.2%. A two sample t-test to compare the lobe(s) with the tumor against the other lobes showed that two patients had significant differences (both *p* < 0.01).

## 4. Discussion

In this paper, we analyzed the lung tissue functional heterogeneity on an interlobar and intralobar basis that could be useful for decision making in pulmonary surgeries or planning in radiation therapy. We constructed 5DCT models to obtain end-exhalation and end-inhalation scans that served as the basis for all calculations. We used the scans and the deformation vectors mapping them to estimate elasticity using a validated biomechanical model. Heterogeneity was analyzed across lobes as well as within lobes. Since heterogeneity was consistently observed in elasticity at both levels and was dependent on COPD in more severe cases, we conclude that the heterogeneity of lung function warrants further investigation to improve surgical decision making and radiotherapy planning.

Elasticity offers a biomechanical property to indicate lung tissue functionality. Though the biomechanical model used in this study takes a DVF from image registration as input, the iterative process calculated a model-based DVF as elasticity is updated. This iteration continues until the DVF converges to the registration-based DVF. However, since the model-based DVF is calculated from the governing equations (Equations 2–4), small, nonphysical errors in the original DVF will be regularized by the iterative approach. This is an advantage over gradient-based ventilation techniques because detectable registration errors will be less likely to impact elasticity. However, a holistic approach to characterizing lung function should include elasticity and ventilation as complements to offer a more comprehensive picture of lung function. Our future work will include a focus on developing a reliable and validated ventilation calculation technique. Once this is realized, we will perform a thorough comparative study between elasticity and ventilation in terms of heterogeneity, impact of COPD, and how to interpret the different physiology represented by each. Once registration techniques improve in accuracy and robustness, and a dedicated comparative analysis is performed, the combination of these metrics will make for a very powerful tool in medicine.

The 5DCT approach used in this study has been well-validated in accurately modeling tissue motion. However, the accuracy of the modeling still has two primary limitations. One is motion blur. Though mitigated by the fast-scanning protocol, some motion blur artifacts may persist. The second limitation is from image registration. The registration technique used has been well-validated and shown to produce very accurate results. Based on a TG-132-based (52) analysis of the registrations used in this study, we calculated the target registration error of 50 manually defined anatomical landmarks per patient to be 1.31 ± 0.87 mm on average. In some instances, some inaccuracies were still found in the inferior lungs possibly due to blur in the images as well as the need to register larger deformations. In the future, the motion blur correction and improvements in image registration techniques should ameliorate these issues, respectively. Finally, more detailed model terms such as a cardiac motion term could be included in future developments to fine tune model accuracy.

Lobe segmentation was performed using open-source software with published validation (48). However, in certain cases, minor corrections were required possibly due to fissure incompleteness. To reduce segmentation time and increase accuracy, a recently published machine learning technique (53) will be implemented in future work. Additionally, the vessels were segmented using a threshold technique. There may be noise in the HU and boundary voxels that could cause some parenchymal voxels to be mistakenly segmented out of the images. Therefore, in the future, we will incorporate blood vessel tracking algorithms to increase the sophistication of the technique and maintain as many parenchymal voxels as possible.

The tissue elasticity estimation using YM has been well-validated in previous studies (34). However, inaccuracies in the images or DVFs could potentially limit the model. Future work will include using a machine learning approach to estimate elasticity from just the end-exhalation scans, which would help to limit the effects of DVF inaccuracies (50). In the future, this will replace the current iterative approach for faster and more accurate results. This technique could greatly reduce the computation time (from several days per patient to the order of a few seconds). This would enable faster data collection and processing as well as a clinical path to relevance for tissue elasticity estimation.

Limitations of the study results are largely derived from the retrospective data collection. For example, patient information like spirometry-derived function data and smoking history could have provided a more holistic explanation for tissue elasticity in these patients, especially patients with no COPD but an extensive smoking history. In the future, with a prospective protocol, we will acquire consistent spirometry function measurements before or after 5DCT acquisition to compare our results to a more quantitative assessment of COPD as well as carefully documented smoking history. Additionally, our sample size is a major limitation in this study. With small numbers of patients in each COPD severity group, our conclusions are interesting, but mostly hypothesis generating, and we will require a larger cohort in our next studies to continue investigating the relationship between COPD and elasticity heterogeneity. Furthermore, our statistical testing is limited as well by the small cohort size. However, if we use a Mann–Whitney *U*-test instead of the *t*-test, which offers a nonparametric alternative with fewer assumptions about the distribution (54), our findings remain the same. Even still, future studies will require additional patients in each COPD severity group.

In this study, we chose to investigate elasticity as it related to physician-determined COPD status. Parametric response mapping is a quantitative alternative to assessing COPD status that categorizes voxels based on thresholds in deep inspiration or expiration scans (55, 56). However, in our study, we used 5DCT-based deformations calculated from free-breathing CT and thus did not have the required adjusted HUs to perform this analysis. Moreover, new parametric response mapping category definitions would need to be determined to assess quiet respiration scans. Another planned development of our technique is to work towards incorporating these approaches to capture a more detailed comparison of voxel-specific disease to elasticity.

In conclusion, heterogeneity of tissue elasticity was consistently observed within and across lobes in patients with no COPD, mild COPD, and moderate-to-severe COPD. Increased heterogeneity was observed with patients with moderate-to-severe COPD. Therefore, elasticity measurement on a lobar or sublobar basis could enable the best guidance for decision making during function sparing treatment planning.

## Data availability statement

The raw data supporting the conclusions of this article will be made available by the authors, without undue reservation.

## Ethics statement

The studies involving human participants were reviewed and approved by Institutional Review Board IRB #19-002033. Written informed consent for participation was not required for this study in accordance with the national legislation and the institutional requirements.

## Author contributions

ML, DL, and AS: research design. DL, DO’C, and AR: 5DCT development and data collection. MM-G: general study design and radiology context. AS and BS: tissue elasticity development and data processing. JG and IB: pulmonology expertise and guidance during heterogeneity analysis. ML and BS: data processing and analysis. ML, BS, and LN: drafting and critically revising the paper. All authors contributed to the article and approved the submitted version.
